# A rare case of bilateral para-rectal hydatid disease: A case report

**DOI:** 10.1016/j.ijscr.2023.108388

**Published:** 2023-06-09

**Authors:** Oussama G. Nasrallah, Arwa ElRifai, Shafik Sidani, Faek Jamali

**Affiliations:** a Division of Urology, Department of Surgery, American University of Beirut Medical Center, Beirut, Lebanon; b Department of Surgery, American University of Beirut Medical Center, Beirut, Lebanon; c Faculty of Medicine, American University of Beirut Medical Center, Beirut, Lebanon

**Keywords:** Pelvis, Para-rectal, Echinococcus, Urticaria, Hydatid

## Abstract

**Introduction:**

Cystic hydatidosis is a parasitic infection caused by the organism Echinococcus Granulosus that is well known to be endemic in the Mediterranean region, eastern Europe and South America and usually presents as hydatid disease of the liver but may affect other organs. The disease occurs when humans become the accidental host through ingestion of the eggs from contaminated food.

**Case presentation:**

We present a case of Hydatid disease presenting as hives refractory to medical therapy over 4 years duration which was revealed to be caused by para-rectal hydatid cysts. Patient received Albendazole for a duration of 2.5 months and then underwent laparoscopic resection of the para-rectal cysts.

**Clinical discussion:**

Pelvic Hydatidosis is a very rare condition accounting for 0.7 % of all cases reported. In most cases, it is coexistent with cysts present elsewhere in the body, namely the liver, which is the case in the presented patient. Imaging is used as a modality to establish the diagnosis of cystic hydatidosis namely Ultrasound (US), Computerized Tomography (CT) and Magnetic Resonance Imaging (MRI). The incidental finding of the hydatid cysts in this patient demonstrated the efficiency of a CT scan as a tool for detection and subsequently diagnosis of the disease in the pelvis. Surgery is the treatment of choice for cysts with daughter vesicles that are not candidates for percutaneous drainage, large liver hydatid cysts of more than 10 cm in diameter, cysts with a risk of rupture in case of trauma, and extrahepatic disease such as the lung, bone, brain, kidneys or pelvis.

**Conclusion:**

This article reports the rare occurrence of para-rectal hydatid disease which is only described in few case reports in the literature and provides an overview on diagnosis, and management of the disease.

## Highlights

• Hydatid disease is endemic in the Mediterranean region, eastern Europe and South America usually affecting the liver.
• Hives may be a presentation of Hydatid disease.
• CT scan detects liver and concomitant para-rectal hydatid cysts.
• Indirect Hemaglutination Assay is used as Serologic evaluation of hydatid disease.
• Albendazole is a standard treatment prior to laparoscopic resection.

## 1 Introduction

Cystic hydatidosis is a parasitic infection that is well known to be endemic in the Mediterranean region, eastern Europe and South America and usually presents as hydatid disease of the liver but may affect other organs [1]. It is caused by the larval stage of the parasite Echinococcus Granulosus, which has maintained its existence through a dog-sheep cycle: the dog (the definitive host) ingests the cyst infested organs of the sheep (the intermediate host) and defecates the eggs of that organism in the wilderness and sheep are re-infected as they graze upon the soiled grass. The disease occurs when humans become the accidental host through ingestion of the eggs from contaminated food [2]. This cystic infection can develop in any organ in the human body. Some organs however are more commonly involved than others such as the liver (60–70 %), the lungs (25 %), the kidneys (3–4 %), the brain (2–3 %). Other organs account for a very small percentage of reported cases [[1], [2], [3]]. We report a case rare case of bilateral para-rectal hydatid disease with concomitant involvement of the liver.

The work has been reported in line with the SCARE criteria [4].

## 2 Presentation of case

**Fig. 1:**
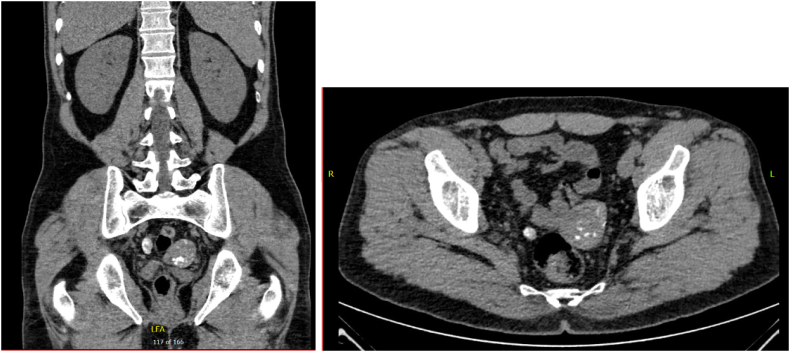
CT showing the 4.9 × 4 cm left pararectal cyst and the 1.5 × 1 cm right pararectal cyst.

**Fig. 2:**
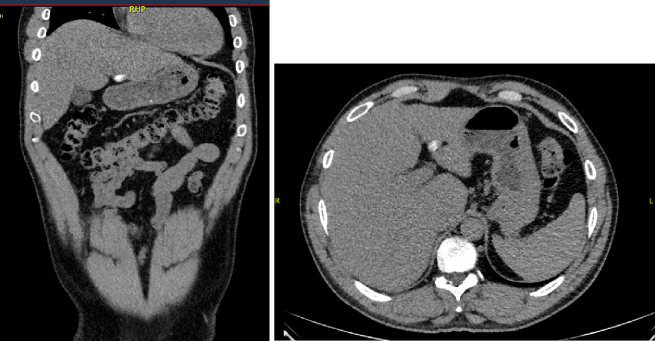
CT showing calcified cyst in segment III of the liver.

This is a case of a 46-year-old male, previously healthy, who previously presented 4 years ago with a 3-month duration of hives which responded to antihistamines. However, his symptoms increased in severity which required additional medical management including Montelukast, a Leukotriene Receptor Antagonist, and Omalizumab, an Anti-IgE monoclonal antibody. The patient underwent a CT scan of the abdomen and pelvis 8 months prior to the discovery of his condition for an episode of flank pain to rule out nephrolithiasis. It showed three calcified lesions raising suspicion for Hydatidosis: a 4.9 × 4 cm calcified mass in the pelvis to the left side of the rectum just superior to the left seminal vesicle causing a mass effect on the adjacent pelvic structures, a 1.5 × 1.1 cm lesion to the right side of the rectum (Fig. 1), and a 1.1 × 0.9 cm calcified lesion in segment III of the liver (Fig. 2). CT scan of the chest was done which showed no evidence of cysts.

Serologic and tissue investigations were done to characterize the lesions: Echinococcus Granulosus Indirect Hemaglutination Assay showed a titer of 1:32 and a CT-guided core biopsy of left pelvic mass showed laminated membranes with calcifications highly suggestive of the diagnosis of Hydatid cyst. Stool cultures were taken and showed no ova or parasites.

**Fig. 3:**
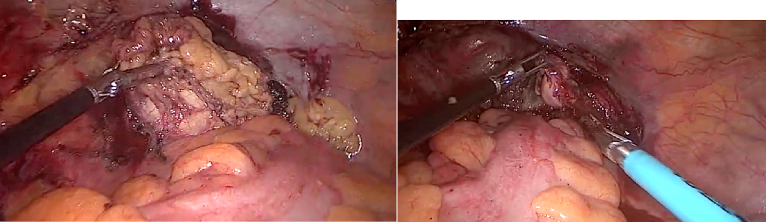
Intraoperative picture of pelvic hydatid cyst after complete mobilization.

Patient received a course of Albendazole therapy (400 mg twice daily) for 2.5 months and was reevaluated by CT scan of the abdomen and pelvis which re-demonstrated the three unchanged cystic lesions in the pelvis and liver. Patient had no signs of fever or chills denied any episodes of nausea or vomiting however he reports having persistent hives. Patient then underwent Laparoscopic en-bloc resection of the para-rectal hydatid cysts with part of the abutting omentum without cyst rupture (Fig. 3). Throughout the surgery, the pelvis was irrigated with Cetrimide. The cyst in the liver was not excised because it was calcified and inactive. The specimen was sent to pathology and confirmed the cystic hydatidosis. Postoperative period was uneventful and the patient was discharged day 1 post-operatively.

## 3 Discussion

Hydatid disease is a zoonotic infection characterized by the formation of cysts that are caused by the accidental ingestion of the egg stage of the tapeworm Echinococcus Granulosus (*E. granulosus*). The medical literature has recognized two species that cause the disease in humans which are: *E. granulosus* and *E. multilocularis*. The layer of the formed cyst called the pericyst is composed of fibrous tissues that become compressed and calcified over time. Whereas, the inner layers form a laminated membrane and germinal center, referred to as the endocyst, which hold the larval stage and may contain daughter cysts. The infection can spread to other organs, or to other parts of the same organ, thereby having secondary echinococcosis [5].

Pelvic Hydatidosis is a very rare condition accounting for 0.7 % of all cases reported [6]. In most cases, it is coexistent with cysts present elsewhere in the body, namely the liver, which is the case in this patient. A hydatid cyst that is present in the pelvis causing mass effect on adjacent structures such as the rectum, bladder, seminal vesicles or the vas deferens could cause a variety of symptoms which include lower urinary tract symptoms, hematospermia, obstructive azoospermia, or constipation [7]. The patient had no symptoms related to the adjacent organs were noted.

Imaging is used as a modality to establish the diagnosis of cystic hydatidosis namely Ultrasound (US), Computerized Tomography (CT) and Magnetic Resonance Imaging (MRI). US is mostly useful in detecting cystic membranes, septations, hydatid sand, and allowing the classification of the hydatid cyst into its categories. CT is highly efficient in showing calcifications in the cystic wall. It is the imaging modality of choice to detect seeding in the peritoneal cavity and elsewhere in the body. In addition, CT scan permits accurate detection of osseous lesions, however MRI is superior in demonstrating neural involvement, and the local extent of the lesion in soft tissues [8]. The incidental finding of the hydatid cysts in this patient demonstrated the efficiency of a CT scan as a tool for detection and subsequently diagnosis of the disease in the pelvis.

Approaches to treating hydatid disease are through medical, percutaneous, or surgical techniques. Albendazole can be used in the management of small cysts without septations and a diameter less than 5 cm as initial management with a maximum dose of 400 mg orally twice daily. However, patients with chronic liver disease or bone marrow suppression should not be treated with Albendazole due to hepatotoxic effect of the drug and the reported risk of cytopenia and agranulocytosis. It is an appropriate therapy for patients who cannot undergo percutaneous treatment or surgery yet it yields significantly favorable results when used as adjuvant therapy by decreasing the risk of recurrence by inactivation of protoscolices and increasing the success rate of the surgery by an odds ratio of 48 (95 % CI: 4–586) as found in a meta-analysis by Velasco-Tirado et al. [9,10]. In terms of duration of treatment, there is no clear recommendation to the specific duration that Albendazole should be administered. However there is a consensus on a duration of 1 to 3 months pre-operatively followed by 1 to 3 months post-operatively [10,11].

Surgery is the treatment of choice for cysts with daughter vesicles that are not candidates for percutaneous drainage, large liver hydatid cysts of more than 10 cm in diameter, cysts with a risk of rupture in case of trauma, and extrahepatic disease such as the lung, bone, brain, kidneys or pelvis [12]. Laparoscopic surgery can be used as a treatment for cystic hydatidosis although there has not been any randomized clinical trials comparing it to open procedures. It has been however associated with increased risk of spillage due to the increase intra-abdominal pressure intraoperatively by pneumoperitoneum [13]. In this patient, no spillage has occurred during the resection of the pelvic cysts and the pelvis was flooded with Cetrimide for infection prophylaxis. If spillage in the peritoneum occurs, the peritoneal cavity should be washed with hypertonic saline and the patient should be treated with albendazole for 3–6 months after the surgery to prevent seeding of the parasite, and a brief course of praziquantel should be administered in addition to the management of anaphylaxis [14].

It is known that hydatid disease may involve the pelvis and its various contents: the bladder, the seminal vesicles, the sigmoid colon and the rectum. There are few case reports published which describe retrovesical hydatid disease which presenting with various lower urinary tract symptoms in males [[15], [16], [17]], and ovarian hydatid disease which may mimic endometriosis as reported by Bozkurt et al. in their case report [18]. Yet para-rectal hydatid disease remains rare and few case reports have been published in the literature: there was concomitant involvement of the ovary and the para-rectal space in the article of Bozkurt et al. [18], a reported ischioanal fossa hydatid cyst was reported by Abdalla et al. [19], and cases of primary hydatidosis of the gluteus muscle causing a rectal shift reported by Charalambous et al. [20].

## 4 Conclusion

In conclusion, pelvic hydatidosis is a rare finding and is more commonly found in areas where zoonotic infections are endemic. Pararectal hydatid disease is described in only a few case reports in the literature. Hydatid disease should be considered in the differential diagnosis of a patient with chronic urticaria, and eosinophilia. The imaging modality for diagnosis of Liver Hydatidosis is Ultrasound, but CT can be used for detection of extrahepatic involvement of the disease specially for calcified lesions. Surgery remains the most used and reliable treatment of Echinococcosis, with Adjuvant Albendazole therapy 1 to 3 months prior to surgery and 1 to 3 months after surgery to prevent recurrence. Hydatid cysts that are large, septated, extrahepatic and that have a high risk of rupture should receive surgical management. However, stable and inactive calcified cysts, should be observed, and followed upon by Ultrasound or CT scan depending on their location. Lastly, small cysts having a single compartment and a diameter less than 5 cm can be treated with Albendazole only with monitoring and follow up in clinic.

## Ethical approval

As this publication is a case report that contains no identifiable content to the patient, this publication was exempt from ethical approval by the Human Research Protection Program (HRPP) and its Institutional Review Board (IRB) at the American University of Beirut Medical Center, Beirut, Lebanon.

## Sources of funding

N/A

## Guarantor

Oussama Nasralla, MD

Division of Urology, Department of Surgery, American University of Beirut Medical Center, Beirut, Lebanon

## Registration of research studies

n/a

## Consent

Written informed consent was obtained from the patient for publication of this case report and accompanying images. A copy of the written consent is available for review by the Editor-in-Chief of this journal on request.

## CRediT authorship contribution statement

**Oussama G. Nasrallah:** Resources, Writing – original draft. **Arwa ElRifai:** Resources, Writing – original draft, Supervision. **Shafik Sidani:** Writing – review & editing. **Faek Jamali:** Conceptualization, Writing – review & editing, Supervision.

## Conflict of interest statement

N/A
